# Phenotypic and Genotypic Features of a *Salmonella* Heidelberg Strain Isolated in Broilers in Brazil and Their Possible Association to Antibiotics and Short-Chain Organic Acids Resistance and Susceptibility

**DOI:** 10.3389/fvets.2017.00184

**Published:** 2017-11-01

**Authors:** Elizabeth Santin, Ricardo Mitsuo Hayashi, Jessica Caroline Wammes, Ricardo Gonzalez-Esquerra, Marcelo Falsarella Carazzolle, Caio César de Melo Freire, Paulo Sérgio Monzani, Anderson Ferreira da Cunha

**Affiliations:** ^1^Laboratório de Microbiologia e Ornitopatologia, Universidade Federal do Paraná, Curitiba, Brazil; ^2^Novus International, Inc., Indaiatuba, Brazil; ^3^Laboratório de Genômica e Expressão, Universidade Estadual de Campinas, Campinas, Brazil; ^4^Laboratório de Bioquímica e Genética Aplicada, Departamento de Genética e Evolução, Centro de Ciências Biológicas e da Saúde, Universidade Federal de São Carlos, São Carlos, Brazil; ^5^Faculdade de Zootecnia e Engenharia de Alimentos, Departamento de Medicina Veterinária, Universidade de São Paulo, Pirassununga, Brazil

**Keywords:** antibiotic, comparative genomics, organic acids, resistance, UFPR1 strain

## Abstract

*Salmonella enterica* serovar Heidelberg is a human pathogen also found in broilers. A strain (UFPR1) has been associated with field reports of resistance to short-chain organic acids (SCOA) in broilers in the South of Brazil, but was susceptible to a *Bacillus subtilis*-based probiotic added in feed in a related study. This work aimed to (i) report clinical symptoms caused by SH UFPR1 in broilers, (ii) study its susceptibility to some antibiotics *in vitro*, and (iii) SCOA *in vivo*; and (iv) relate these phenotypic observations with its genome characteristics. Two *in vivo* trials used 1-day-old chicks housed for 21 days in 8 sterilized isolated negative pressure rooms with 4 battery cages of 12 birds each. Birds were challenged or not with 10^7^ CFU/bird of SH UFPR1 orally and exposed or not to SCOA in a 2 × 2 factorial design. Zootechnical parameters were unaffected (*P* > 0.05), no clinical signs were observed, and few cecal and hepatic histologic and immune-related alterations were seen, in birds challenged with SH. Formic and propionic acids added together in drinking water, fumaric and benzoic acid in feed (Trial 1), and coated calcium butyrate in feed (Trial 2) did not reduce the SH isolation frequencies seen in cecum and liver in broilers after SH challenge (*P* > 0.05). SH UFPR1 was susceptible to amikacin, amoxicillin + clavulanate, ceftiofur, cephalexin, doxycycline and oxytetracycline; and mildly susceptible to ampicillin + sulbactam, cephalothin, ciprofloxacin, enrofloxacin, and gentamycin in an *in vitro* minimum inhibitory concentration model using Mueller–Hinton agar. The whole genome of SH UFPR1 was sequenced and consisted of a circular chromosome, spanning 4,760,321 bp with 52.18% of GC-content encoding 84 tRNA, 22 rRNA, and 4,427 protein-coding genes. The comparison between SH UFPR1 genome and a multidrug-resistant SL476 strain revealed 11 missing genomic fragments and 5 insertions related to *bgt, bgr*, and *rpoS* genes. The deleted genes codify proteins associated with cell cycle regulation, virulence, drug resistance, cellular adhesion, and salt efflux which collectively reveal key aspects of the evolution and adaptation of SH strains such as organic acids resistance and antibiotic sensitivity and provide information relevant to the control of SH in poultry.

## Introduction

Despite recent advances in the treatment of infectious diseases, pathogenic microorganisms, including *Salmonella*, remain an important threat to human and animal health worldwide (1). Non-typhoid *Salmonella* serovars are well-known pathogens but they also silently infect animals, particularly poultry, as transient members of the intestinal microbial population without causing disease (2, 3). Intestinal colonization of several *Salmonella enterica* serovars often have no effect on poultry zootechnical performance (3). Kogut et al. (4) described that the infection in chickens caused by *Salmonella* Enteritidis leads to immune tolerance beginning around 3–4 days post-primary infection. This decreases the host immune responsiveness resulting in the establishment of *Salmonella* and persistent colonization. This asymptomatic infection could increase the probability of transmission to humans *via* contaminated food (5).

Therefore, reducing *Salmonella* colonization and fecal shedding in live chickens, and its subsequent chicken meat contamination, can reduce the burden of salmonellosis in humans. Although many aspects related to their mechanisms of action are unknown, short-chain organic acids (SCOA) have been added to chicken feed, drinking water, and other matrices, as part of several strategies to prevent *Salmonella* colonization in animal tissues and transmission through the food chain with many positives results (6).

In that context, *Salmonella enterica* serovar Heidelberg is one of the most prevalent serovars and is commonly isolated from patients with salmonellosis in North America. That region has the greatest prevalence comparing to other continents (7). More invasive human infections, such as myocarditis and bacteremia, are produced by SH compared to other non-typhoid *Salmonella* (8). Since 1962, SH has been isolated and reported from poultry and their products worldwide (9) including Brazil (10). Voss-Rech et al. (11) reported 20 different *Salmonella* serovars in samples from broilers and SH was prevalent in 7.31% of them. SH UFPR1 strain was isolated from a chicken carcass in the South of Brazil. This strain showed susceptibility and resistance to antibiotics and organic acids, respectively. In a previous trial, our group showed reduction in SH counts in the cecum and liver of broilers fed with a probiotic composed of three strains of *Bacillus subtillis* previously challenged orally with SH UFPR1 strain. No studies comparing the genome of Brazilian UFPR1 to other SH strains have been reported.

Whole-genome sequencing is a tool that allows to investigate the genomic features of any organism. Several genomes from *Salmonella* strains have been decoded using this technique, aiming at improving the understanding of some aspects of their evolutionary biology, distinguishing outbreak-related strains of sporadic infections (12) and comparing genomes of strains with different clinical history and resistance profile (1, 13, 14). Whole-genome sequencing was recently used to study the differences among SH serotypes (15, 16) and resistance to different antibiotics (17, 18).

The objective of this work was to report possible clinical symptoms caused by SH UFPR1 strain in broilers, test the efficacy of SCOA to reduce SH UFPR1 strain proliferation in broilers, study the susceptibility of this strain to some antibiotics *in vitro*, and relate these phenotypic observations with its genome characteristics through comparative genomics.

## Materials and Methods

### *In Vivo* Experiments

The SH UFPR1 strain used herein was isolated from commercial broiler carcasses in the South of Brazil according to the Brazilian Ministry of Agriculture’s procedures [MAPA (19)] and sent to Fiocruz Institute (protocol number 6830/2012) for further serological identification.

Two experiments were conducted at CERIA (Center of Immune Response in Poultry) at the Federal University of Parana, Curitiba, Brazil, to evaluate the effectiveness of several SCOA to control UFPR1 in broilers, and report possible clinical symptoms in orally challenged birds. In Trial 1, an SCOA blend (30% of formic acid and 18% of propionic acid) was offered in drinking water at 0.05% from 1 to 7 and from 15 to 21 days of age, along with 3 kg/ton of feed of a SCOA blend composed of fumaric and benzoic acids at 92% and fed from 1 to 21 days of age. In Trial 2, the effect of adding a product containing 89% of coated calcium butyrate when added at 2 kg/ton in feed from 1 to 21 days was studied.

All experimental procedures were approved by the Ethical Committee of Agricultural Sector of Federal University of Parana under approval number 037/2016 and 014/2016, respectively. All other methods were equal for both trials as described below.

### Chicken Housing and Sample Collection

Eight previously disinfected negative-pressured isolation rooms were used. Rooms were equipped with automatic temperature and lighting controls, and each contained four battery cages (replications) stacked vertically with sterilized litter and nipple drinkers. Before the start of each trial, swabs from all walls and cages within the rooms were collected to verify the absence of *Salmonella* by qualitative analysis. One day-old male chicks (Cobb^®^ 500; *n* = 192) were kept from 1 to 21 days of age and distributed using a completely randomized design of four treatments (*n* = 48 birds per treatment, with four replicates per treatment, 12 birds per replication) detailed as follows: T1: non-challenged plus control diet, T2: non-challenged plus SCOA treatment according to Trial, T3: challenged with SH plus control diet, T4: challenged with SH plus SCOA according to Trial (Table 1).

**Table 1:** Experimental treatment design.

Treatment	Challenge^a^	Short-chain organic acids supplementation^b^
T1: non-challenge (NC)	No	No
T2: NC + short-chain organic acids (SCOA)	No	Yes
T3: SH challenge (SHC)	*Salmonella* Heildelberg UFPR1	No
T4: SHC + SCOA	*Salmonella* Heildelberg UFPR1	Yes

*^a^Challenged orally with 10^7^ CFU/chick. Trial 1 day 1, Trial 2 day 7*.

*^b^In Trial 1, 0.05% of an organic acids blend (30% of formic acid and 18% of propionic acid) offered in drinking water from 1 to 7 days and from 15 to 21 days of age, associated with a treatment with 3 kg/ton of a product with minimum 92% of fumaric and benzoic acids in feed, from 1 to 21 days of age. In Trial 2, a product constituted with coated 89% of calcium butyrate at 2 kg/ton in feed from 1 to 21 days was evaluated*.

At day 1, 10 chicks were necropsied, and liver and cecum samples were collected to confirm the negativity in both *in vivo* experiments by qualitative analysis and at day 21, feed intake (FI), body weight gain (BWG), and feed conversion (FC) were calculated.

At days 7 and 21 in Trial 1 and days 14 and 21 in Trial 2, 12 birds from each treatment were euthanized by cervical dislocation, necropsied and liver and cecum were collected for *Salmonella* sp. counting procedure. In Trial 2, liver and cecum of five birds per treatment were collected for histology at day 14. At that age, mRNA expression of IL-10 and IL-12 was also evaluated in liver.

### Diets

The nutritional value of experimental diets was formulated to supply nutrients at requirements (20). Diets were corn and soybean meal based and were offered in mash form *ad libitum* at all times. Rations were formulated without coccidiostatics or antibiotics and were designed for a unique feeding phase (Starter) from 1 to 21 days of age for all treatments.

A basal diet with all ingredients except amino acids, vitamin, and mineral premix was autoclaved at 120°C/15 min. After this process, SCOA, amino acids, vitamin, and mineral premix were added according to each treatment. All dietary components were mixed for 10 min in a 50 kg blender. Batches were blended in such an order as to avoid interference among treatments.

### *Salmonella* Heidelberg Challenge and Quantification

At 3 or 7 days of age for Trials 1 or 2, respectively, chicks from T3 and T4 were challenged orally with 10^7^ CFU/chick of SH UFPR1.

The quantification of typical colonies of *Salmonella* sp. (quantitative analysis) was performed in liver and cecum samples processed according to the modified methodology by Pickler et al. (21). The organs were weighed, mashed and homogenized in 2% buffered peptone water (1:9). Further dilution was conducted by successively placing 1 mL of the solution in a test tube with 9 mL 0.1% peptone water until a 10^−3^ dilution was achieved. Then, 100 µL aliquots of each dilution were transferred to duplicate plates in Brilliant Green Agar (BGA) medium and uniformly spread with a sterile Drigalsky loop. Plates were incubated at 35°C for 24 h before typical colonies were counted. For all samples, pre-enrichment was performed with 2% buffered peptone water at 35°C for 24 h. Samples that did not show typical *Salmonella* colonies during BGA counting, were enriched with 10 mL Rappaport-Vassiliadis broth and incubated at 42°C for 24 h. Thereafter, a drop of the enriched broth was placed on BGA medium. The samples that were negative after direct BGA plate counting, but positive after enrichment were assumed to have 10^1^ CFU/g. Samples that were negative after enrichment were assumed to have 0 CFU/g. To verify the *Salmonella* serotype, isolated *Salmonella* samples were sent to the Sector of Enterobacteria of the Oswaldo Cruz Institute, Brazil for serotyping.

### Cecal and Hepatic Histologic Evaluation

For trial 2 only, samples of cecum and liver were processed according to Kraieski et al. (22). Tissues embedded in paraffin were cut in 5 µm sections and both later stained with hematoxylin and eosin, and with Alcian Blue for cecum also. Liver samples were examined using 5 fields per bird with a 10× objective and 100× of magnification. Congestion, hydropic degeneration, cell vacuolation, bile-duct proliferation, immune cells infiltration, pericholangitis, and lymphocytic aggregate were observed. The “I See Inside” (ISI) methodology applied herein was described by Santin et al. (23) where an impact factor (IF) was assigned to each type of alteration according to its potential to reduce organ functionality. The basis for these criteria was previous literature review on the relationship of organ functionality and type of lesion, and background research. The IF ranges from 1 to 3, where 3 defines an alteration that impacts organ functionality the most, and 1 the least. For instance, necrosis has the highest IF because the functional capacity of affected cells is totally lost. In addition, an observer score (OS) is assigned to each lesion based on its observed intensity or frequency compared to non-affected organs, during histologic inspection. This evaluation is performed in each organ/tissue per animal and OS values range from score 0 (absence of lesion or frequency), score 1 (alteration up to 25% of the area or observed frequency), score 2 (alteration ranges from 25 to 50% of the area or observed frequency), and score 3 (alteration extent more than 50% of the area or observed frequency). In order to calculate the final ISI Index, the IF of each alteration is multiplied by the respective score number, and the results of all alterations are summed [ISI = Σ (IF × OS)]. The scale ranges from 0 to 39 for the liver.

For cecum samples, 5 fields per bird in 40× objective and 400× of magnification were evaluated, and villus height, villus thickness, presence of erythrocytes, and infiltration of immune cell on lamina propria were measured.

### Cytokines mRNA Expression in Liver (Trial 2)

Six birds per treatment were euthanized, their livers removed and immediately stored at −20°C until further analysis. Total RNA from that tissue was isolated using Trizol reagent (15596-018, Invitrogen, Carlsbad, CA, USA) following the manufacturer’s procedures. Turbo-DNAse kit (AM1907, Applied Biosystems, Foster City, CA, USA) was used for the collected samples. RNA concentrations were quantified by NanoDrop Spectrophotometer (ND1000, Thermo Scientific, Bonn, Germany) and RNA integrity determined by Experion Automated Electrophoresis System (700-7000, Bio-Rad, Hercules, CA, USA). RNA samples were reverse transcribed and RT-qPCR analyses performed with a MyiQ System (170-9740, Bio-Rad). One microgram of RNA was converted to cDNA in a 20 µL reaction volume using the iScript™ Reverse Transcription Supermix kit (170-8841, Bio-rad) at 25°C for 1 h, 42°C for 30 min, and then 85°C for 5 min.

The genes analyzed by RT-qPCR were: IL-10 (5′-cgggagctgagggtgaa-3′ and 5′-gtgaagaagcggtgacagc-3′), IL-12 (5′-agactccaatgggcaaatga-3′ and 5′-ctcttcggcaaatggacagt-3′), and GAPDH (5′-ggtggtgctaagcgtgttat-3′ and 5′-acctctgtcatctctccaca-3′). The final 20 µL PCR contained 2 µL reverse transcription product, 2 µL of the forward and reverse gene, and 10 µL of iTAq^®^ Universal SYBR Green Supermix (172-5122, Bio-Rad). PCR cycle conditions of all primer pairs used an initial 60 s denaturation step at 95°C followed by 40 cycles of denaturation (15 s at 95°C), annealing and extension (30 s at 60°C). The melting profile of each sample was analyzed after every PCR run to confirm PCR product specificity; and later determined by heating samples at 65°C for 30 s and then increasing the temperature at a linear rate of 20°C/s to 95°C while continuously monitoring fluorescence. Sample PCR amplification efficiencies were determined in the log-linear phase with the LinRegPCR program (24). Additionally, the delta–delta equation subtracts sample and reference Ct values from an endogenous control. However, the endogenous control (GAPDH) Ct was affected by treatments in this study (*P* < 0.05) and, therefore, was removed from the equation. All data were normalized to the mRNA level of the control group (group non-challenged and without SCOA) and reported as the fold-change from the reference, which was calculated as E_S_^(40 − Ct Sample)^/E_R_^(40 − Ct Reference)^, where E_S_ and E_R_ are the sample and reference PCR amplification efficiencies, respectively (25).

### Statistical Analysis of *In Vivo* Studies

Data were analyzed using the statistical software Statistix 9. For microbiological analysis, data were evaluated by the Shapiro–Wilk normality test. Parametric data were subjected to analysis of variance (ANOVA) and Tukey’s test (*P* < 0.05), while the Kruskal–Wallis test (*P* < 0.05) was used for non-parametric data (quantitative microbiological data and histology data). The chi-square test was used in microbiological results of presence/absence (qualitative) of *Salmonella* in liver for Trial 2. For zootechnical performance, and immunohistochemistry analysis, data were subjected to ANOVA using a factorial 2 × 2 design.

### *In Vitro* Antimicrobial Susceptibility Tests

The susceptibility of SH UFPR1 against a panel of 12 antimicrobials commonly used in human and veterinary clinics in Brazil was determined by the dilution antimicrobial method using Mueller–Hinton agar after incubation at 37°C for 18–24 h. The minimum inhibitory concentration (MIC) and minimum bactericidal concentration (MBC) results were interpreted in agreement with the interpretative criteria provided by Clinical and Laboratory Standards Institute (26). The 12 antimicrobials tested included amikacin (250 mg/mL), amoxicillin + clavulanate (14 g + 3.5 g/100 mL), ampicillin + sulbactam (1 g + 0.5 g/10 mL), ceftiofur (50 mg/mL), cephalexin (250 mg/5 mL), cephalothin (1 g/10 mL), ciprofloxacin (2 mg/mL), doxycycline (4.6 g/100 mL), enrofloxacin (10 g/100 mL), gentamycin (40 mg/mL), penicillin (6,000,000 UI/15 mL), and tetracycline (20 g/100 mL). The *Escherichia coli* ATCC 25,922 was used as reference strain. The MIC breakpoints were set based on CLSI (26) and FDA (9).

### Genomic Analysis and Comparative Analysis

The isolated SH UFPR1 strain was cultured overnight in liquid LB medium, its genomic DNA extracted using the QIAamp DNA Mini Kit (Qiagen) and quantified using the NanoVue spectrophotomer (GE Healthcare). A total of 70 µg of DNA was sent to the High-Throughput Sequencing Facility at University of North Carolina. The library was prepared using the PacBio’s 20 Kb template prep protocol (PN_100-286-000-06) and it was size-selected by using a range setting of 8,000 bp to 50,000 bp. *De Novo* assembly was performed using PacBio native pipeline (27). Comparative genomic analysis was independently performed with MAUVE v.20150225 (28) and Mummer v.3.23 (29) programs, using the annotated genome of SH strain SL476 as reference (GenBank assembly accession: GCA_000020705.1) (30). The shared genomic fragments between UFPR1 and SL476 were identified with Mummer while the regions with no match between them were identified with a Perl script (available in https://github.com/CaioFreire/Scripts). PROKKA v.1.12 software was used for genome annotation (31) and the circular map was drawn using DNAPlotter v.10.2 (32). The fully sequenced SH UFPR1 genome was deposited at the NCBI genome database under the number CP020101. In addition, missing fragments between each other were found using Megablast (https://blast.ncbi.nlm.nih.gov) and verifying if the gene sequences in these missing fragments were present in other parts of the genome.

## Results

At the *in vivo* trails, SCOA were offered to the birds during the early days of life to allow the gut to adapt to the treatment before the SH challenge. No effect in zootechnical parameters was observed at any time in both trials (*P* > 0.05) either from the adding SCOA in drinking water and/or feed, from challenging birds with SH UFPR1 strain, or from the interaction between these factors (Table 2). It should be mentioned that the primary objective of this work was not measuring performance and that more replicates would be needed to appropriately test possible effects on these parameters.

**Table 2:** Mean ± SD of feed intake (FI), body weight gain (BWG), and feed conversion (FC) during the periods of 1–21 days of age of broilers on Trials 1 and 2 in non-challenged and challenged birds.

	Period	Non-challenged	Challenged	*P*-value*
FI	1–21 days (Trial 1)	1,078.2 ± 19.03	1,088.3 ± 14.74	0.680
	1–21 days (Trial 2)	1,214.1 ± 33.20	1,197.4 ± 17.51	0.727
BWG	1–21 days (Trial 1)	884.98 ± 19.89	914.46 ± 25.32	0.367
	1–21 days (Trial 2)	836.58 ± 23.01	804.50 ± 24.08	0.341
FC	1–21 days (Trial 1)	1.221 ± 0.01	1.198 ± 0.02	0.468
	1–21 days (Trial 2)	1.452 ± 0.04	1.496 ± 0.03	0.499

*Tukey test.

**Figure 1:**
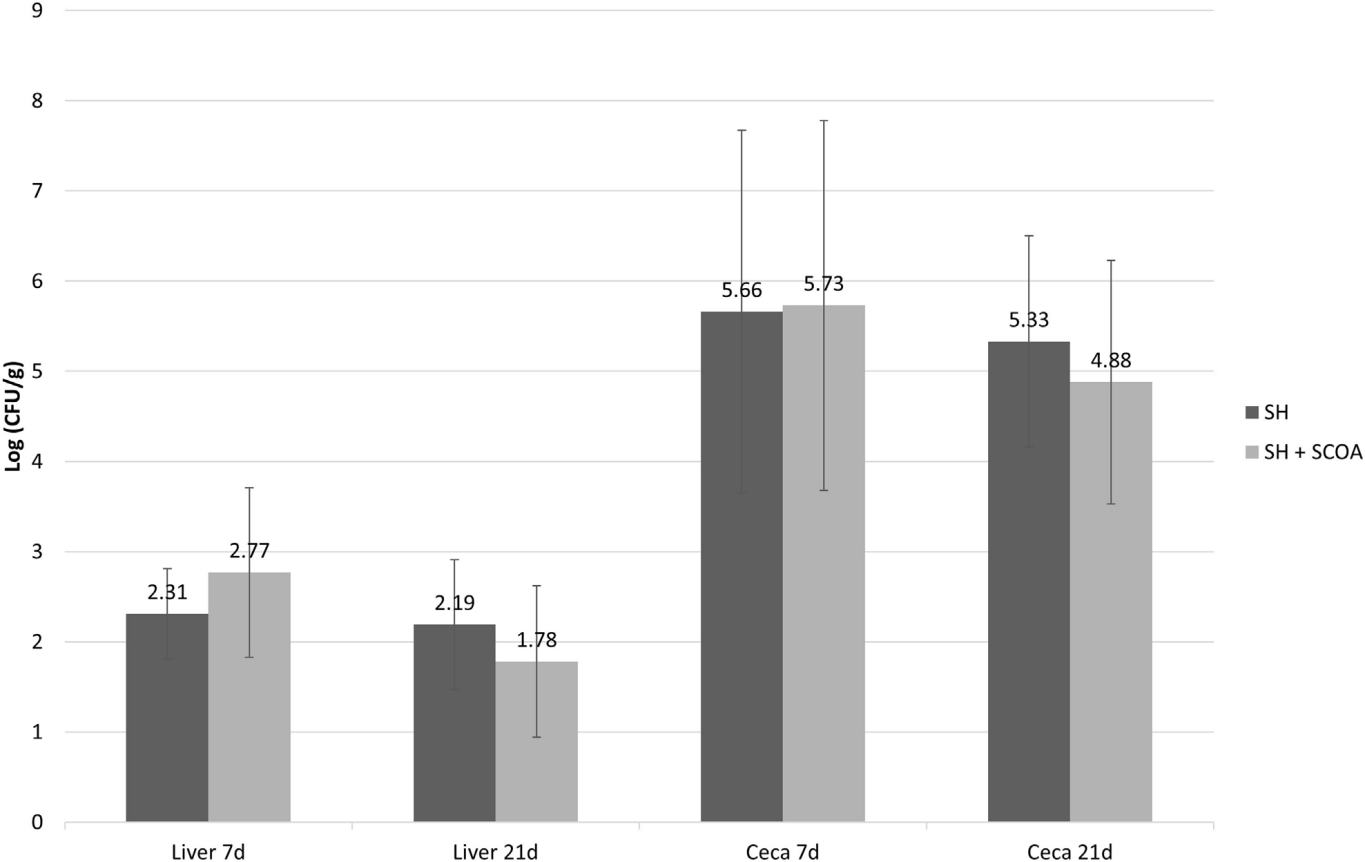
Trial 1. *Salmonella* sp. quantification (Log CFU/g) in liver and cecum at 7 and 21 days of age in treatments challenged with *Salmonella* Heildelberg (SH) or *Salmonella* Heidelberg challenged + Short-Chain Organic Acids (SH + SCOA).

**Figure 2:**
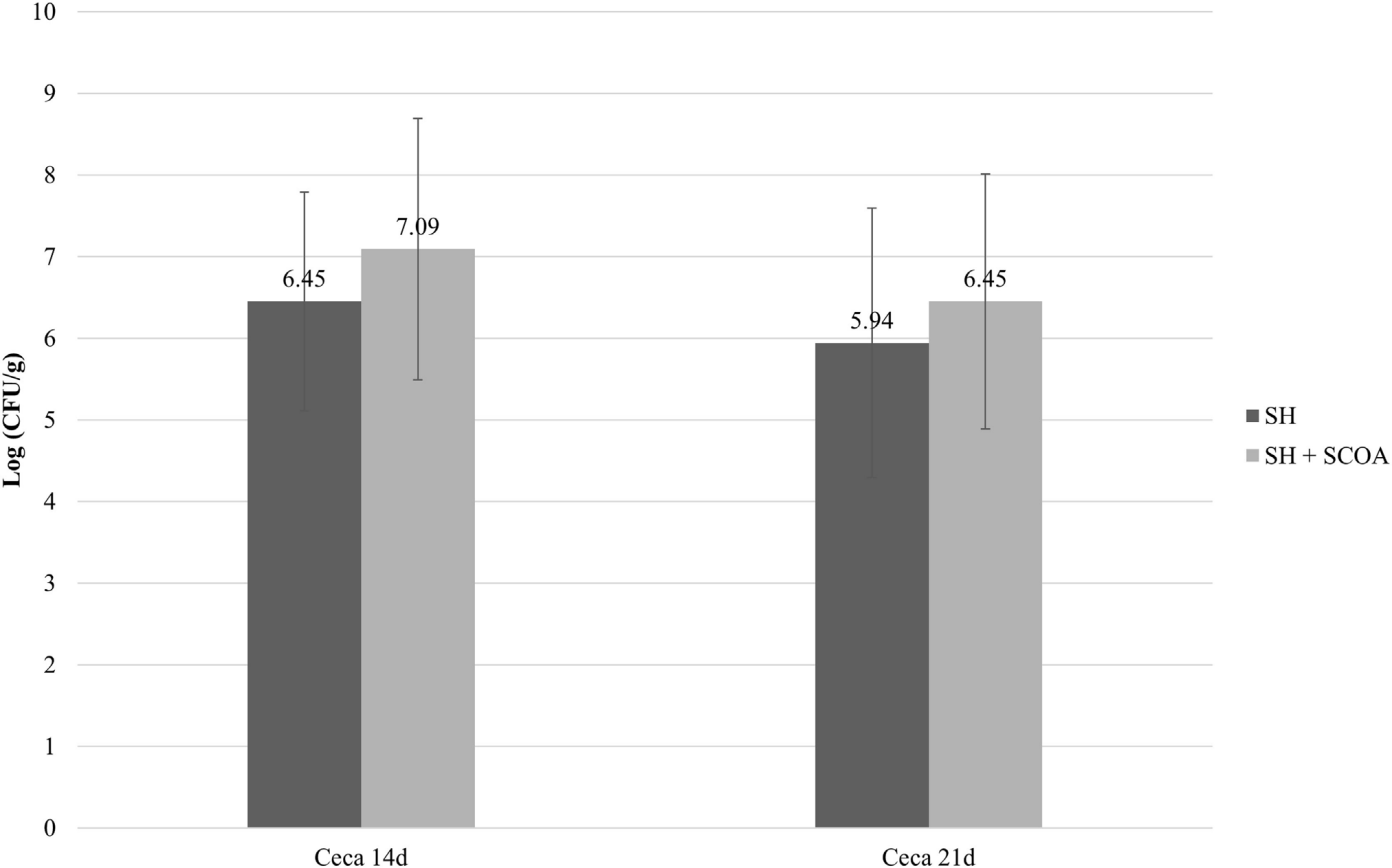
Trial 2. *Salmonella* sp. quantification (Log CFU/g) in cecum at 14 and 21 days of age in treatments challenged with *Salmonella* Heidelberg (SH) or *Salmonella* Heidelberg challenged + Short-Chain Organic Acids (SH + SCOA).

As expected, all non-challenged groups tested negative for *Salmonella* while all challenged groups tested positive. Therefore, data on *Salmonella* counts were statistically evaluated in a completely randomized design using only challenged groups. Still, the use of SCOA did not influence the percentage of SH positive in cecum or liver (Figure 1; Trial 1). Similar findings were observed in Trial 2 in cecum (Figure 2) where SH counts were only performed in that organ. In that experiment, liver microbiological results were qualitative only and showed that, at 14 days of age, 100 and 42% of the samples were SH positive in challenged and challenged + SCOA birds, respectively, while, at 21 days of age, 25 and 58% tested SH positive for those two groups of broilers (*P* < 0.05). When comparing results from both trials, it seems that challenging birds with SH UFPR1 later in life (7 days in Trial 2 vs. 3 days in Trial 1) reduced the recovery of SH in liver.

**Figure 3:**
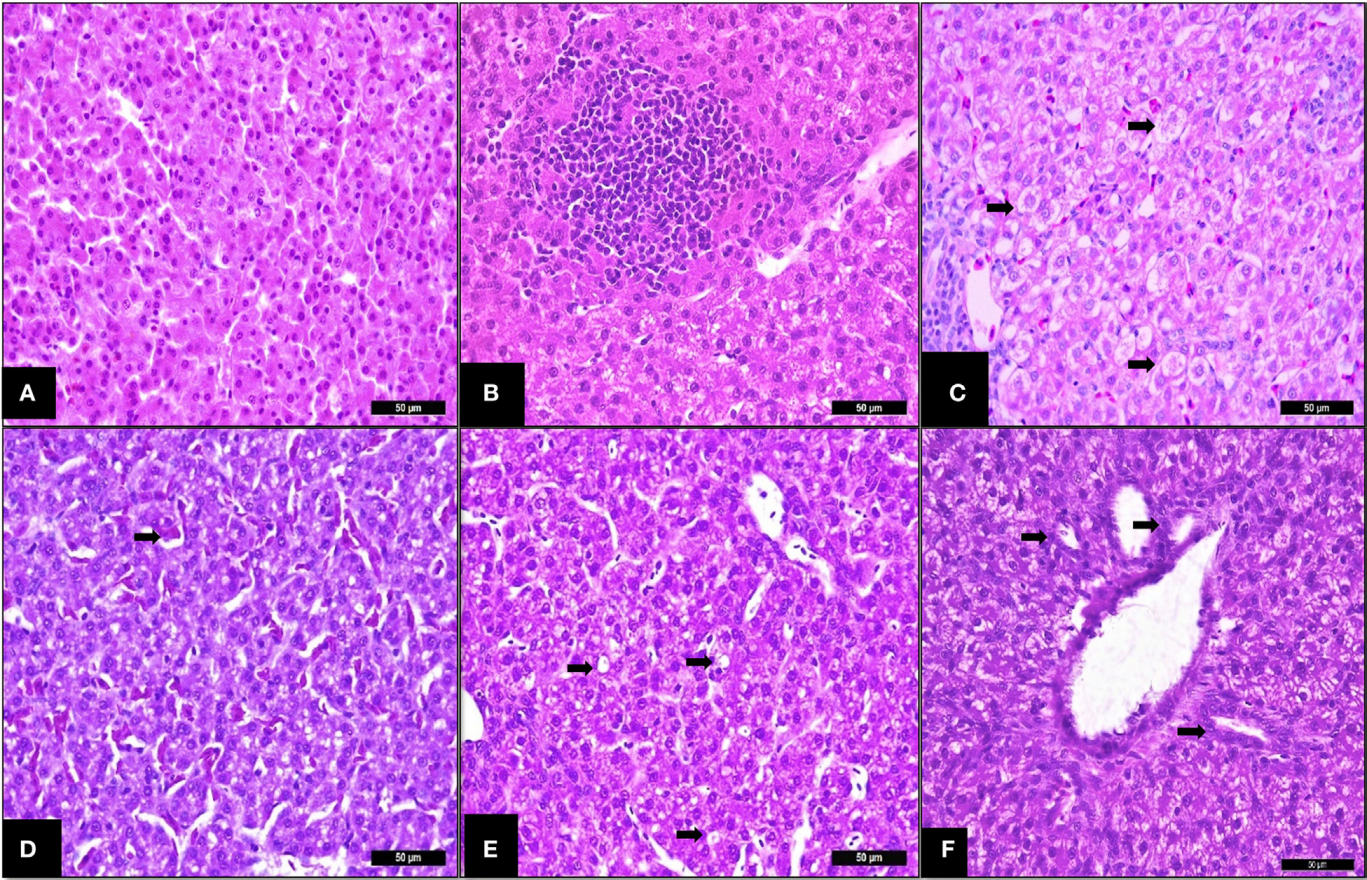
Trial 2. Liver of broilers (14 days). **(A)** Non-challenged group—normal tissue (parenchyma), I See Inside (ISI) score 23. **(B)** SH-Challenged group—ISI score 25, cell infiltrate in parenchyma grade II. **(C)** SH-Challenged group—hydropic degeneration grade III. **(D)** SH-Challenged group—congestion grade II. **(E)** SH-Challenged group—Vacuolization grade II. **(F)** SH-Challenged group—bile-duct proliferation grade II. Hematoxylin and eosin, 400×.

Liver histology revealed that challenging birds with SH increased the ISI score at 14 days indicating greater histological alterations (Table 3). The main alterations found were congestion, vacuolation, and immune cell infiltration as presented in Figure 3. The SCOA treatment did not influence liver histology measures.

**Table 3:** Mean ± SE of histological alterations by I See Inside (ISI) methodology in liver and cecum at 14 days of age, evaluating the challenge and the use of feed additives interaction.

Treatment	ISI liver	Villus height (μm)	Villus thickness (μm)	Area (μm)
Non-challenge	9.93 ± 0.7^b^	164 ± 8.0^b^	91 ± 7.0^b^	14,924 ± 1,591^a^
*S*. Heildelberg Challenge (SHC)	20.31 ± 0.5^a^	176 ± 8.0^a^	108 ± 7.00^a^	19,008 ± 1,591^b^
NC + Short-chain organic acids (SCOA)	12.31 ± 0.7^b^	158 ± 8.0^b^	95 ± 7.00^b^	15,010 ± 1,591^a^
SHC + SCOA	19.10 ± 0.5^a^	175 ± 8.0^a^	106 ± 7.00^a^	18,550 ± 1,591^b^

*^a,b^Different letters in the same row indicate significant difference at P < 0.05*.

**Figure 4:**
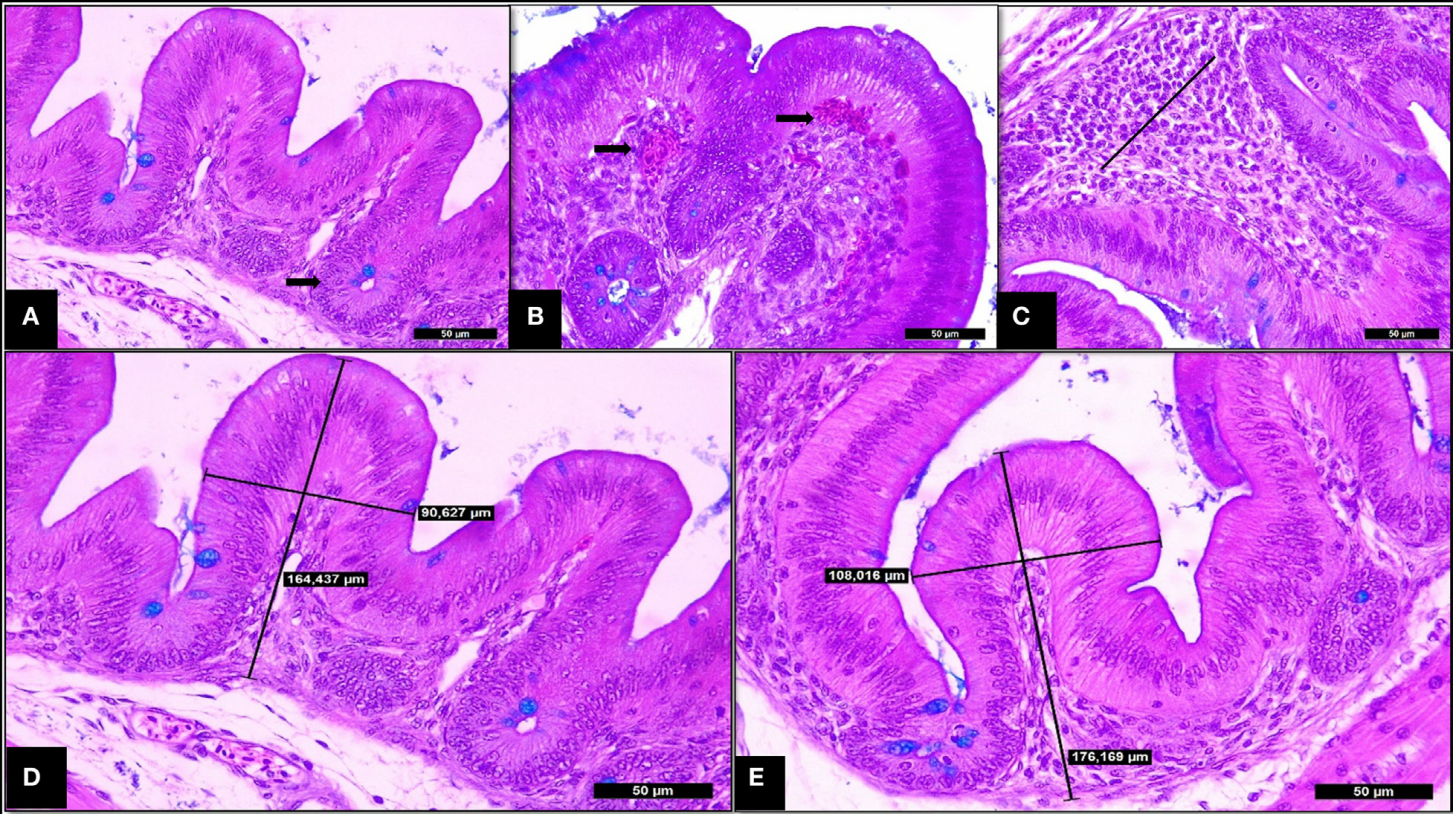
Trial 2. Cecum of broilers (14 days). **(A)** Non-challenged group—normal villi and crypts of Lieberkhün. **(B)** SH-Challenged group—congestion grade III. **(C)** SH-challenged group—cell infiltrate in lamina propria grade II. **(D)** Non-challenged group—villus height and thickness axes of measurement. **(E)** SH-challenged group—villus height and thickness axes of measurement. Hematoxylin and eosin plus Alcian Blue, 400×.

At 14 days of age, SH-challenged birds showed increased cecal villi height, villi thickness and villi surface area compared to the non-challenged group (*P* < 0.05; Table 3; Figure 4). Supplementing SCOA treatment did not affect the histological parameters in cecum having no interaction between SH challenge and SCOA supplementation (*P* > 0.05).

**Figure 5:**
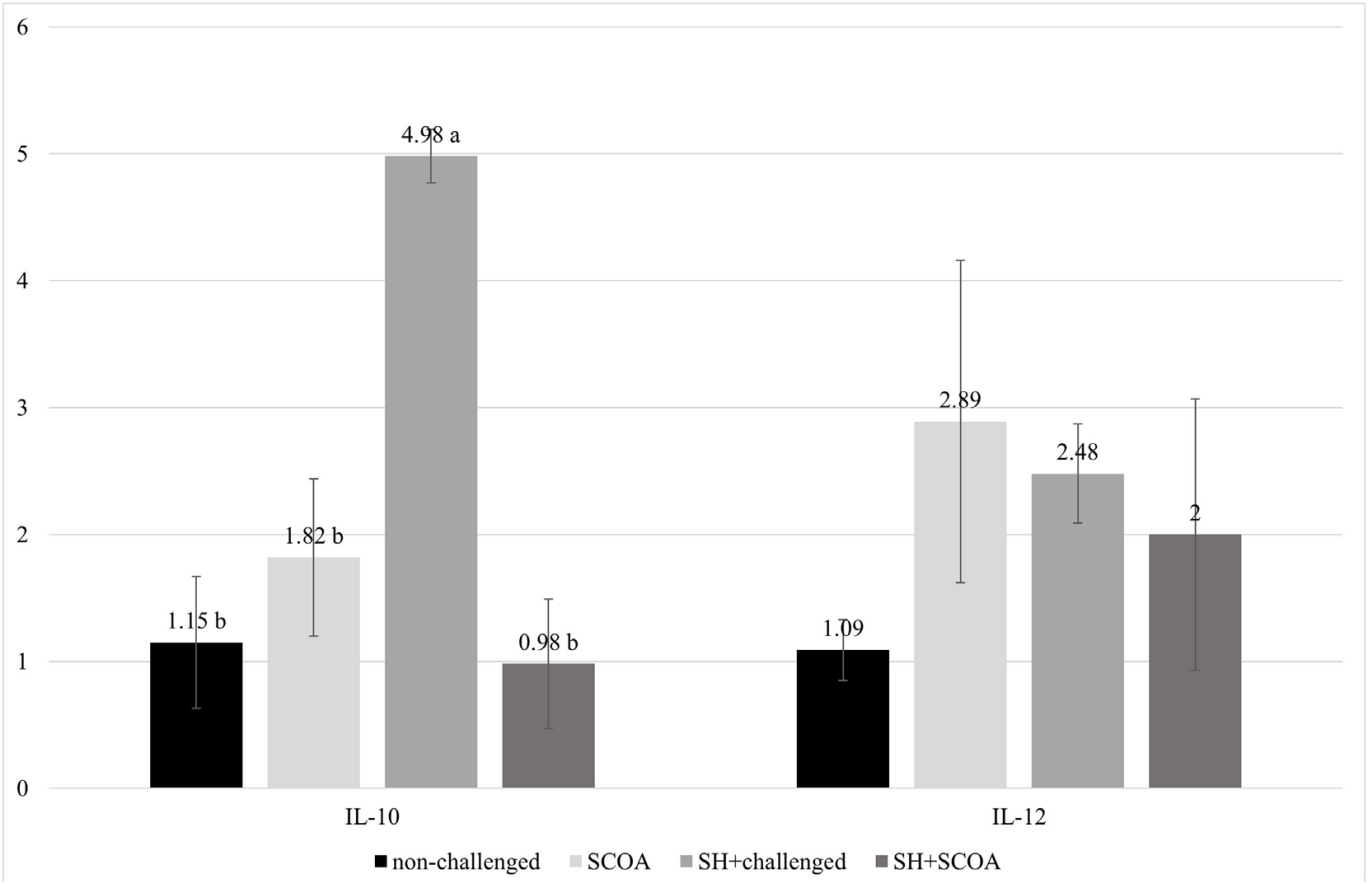
Trial 2. mRNA expression of IL-10 and IL-12 at 14 days of age in non-challenged group, SH-challenged group, short-chain organic acids (SCOA) and SCOA + SH-challenged group. ^a,b^Different letters indicate significant differences at *P* < 0.05 at Kruskal–Wallis test.

The results of mRNA expression of cytokines on liver at 14 days (Figure 5) showed higher IL-10 (*P* > 0.05) cytokines in SH-challenged birds compared to non-challenged, while IL-12 mRNA expression remained unaffected in all treatments.

The *in vitro* work suggests that SH UFPR1 strain is susceptible to amikacin, amoxicillin + clavulanate, ceftiofur, cephalexin, doxycycline and oxytetracycline and presents intermediary resistance to ampicillin + sulbactam, cephalothin, ciprofloxacin, enrofloxacin, and gentamycin (Table 4).

**Table 4:** Minimum inhibitory concentration (MIC) and minimum bactericidal concentration (MBC) of Brazilian *Salmonella enterica* serovar Heidelberg (UFPR1 strain).

Antibiotic	MIC (μg/mL)	MBC (μg/mL)	Breakpoint
Amikacin	1.90	61	Susceptible
Amoxicillin + clavulanate	≤0.06 + 0,15	875,000 + 218,750	Susceptible
Ampicillin + Sulbactam	24.41 + 3.05	6,250	Intermediate
Ceftiofur	1.52	25,000	Susceptible
Cephalexin	0.7625	–	Susceptible
Cephalothin	24.41	50,000	Intermediate
Ciprofloxacin	0.24	–	Intermediate
Doxycycline	1.40	718.75	Susceptible
Enrofloxacin	0.76	3,125	Intermediate
Gentamycin	1.22	9	Intermediate
Oxytetracycline	1.64	13,500	Susceptible

*Inoculum SH UFPR1 strain: 2 × 10^8^ CFU/mL*.

**Figure 6:**
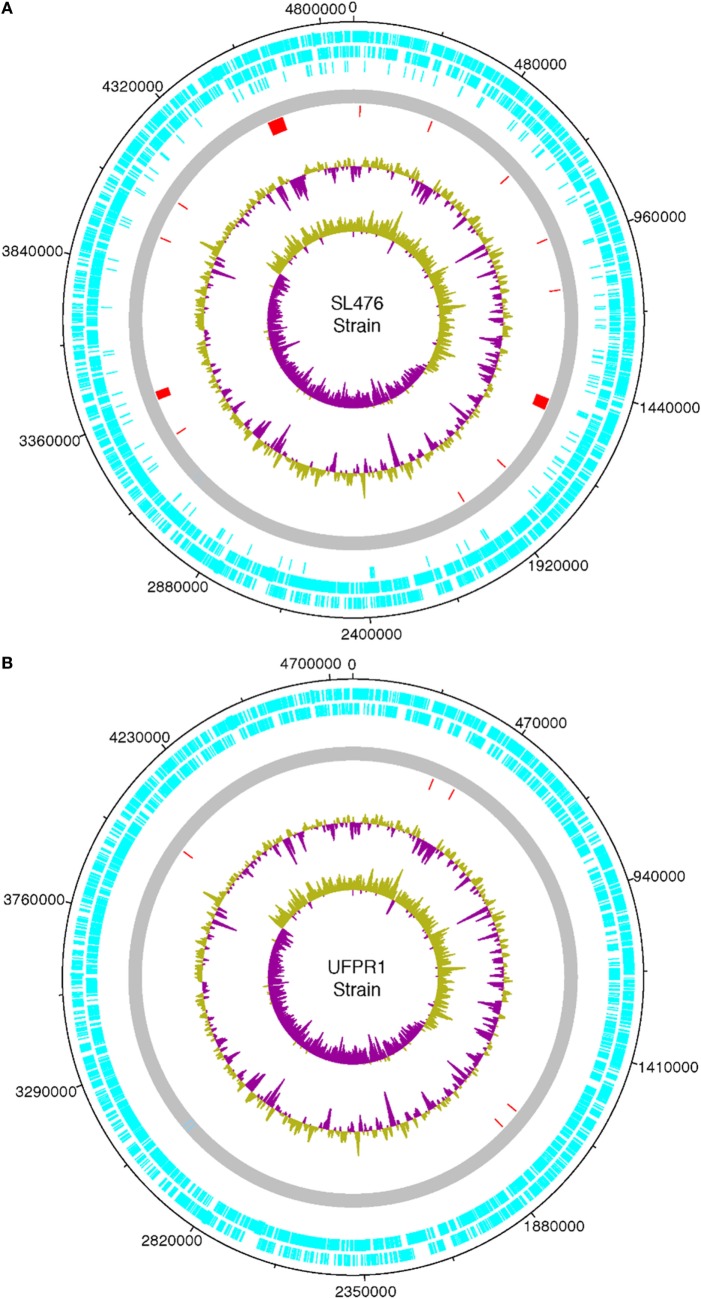
Chromosome features of a Brazilian UFPR1 strain **(B)** compared to SL476 strain **(A)** isolate. The circular map was drawn using DNA Plotter. Different features are shown in different colored bars. The coding sequences are shown in light blue (forward and reverse). The complete genome is shown in gray, the red dashes represents unique chromosome regions that have no homologous sequence in the genome of the other strain, green and purple in the major circle represent the GC content, while in the central circle show the GC skew [(G − C)/G + C]. Regions with GC content below the average are shown in purple and those with content above the average are shown in green.

**Figure 7:**
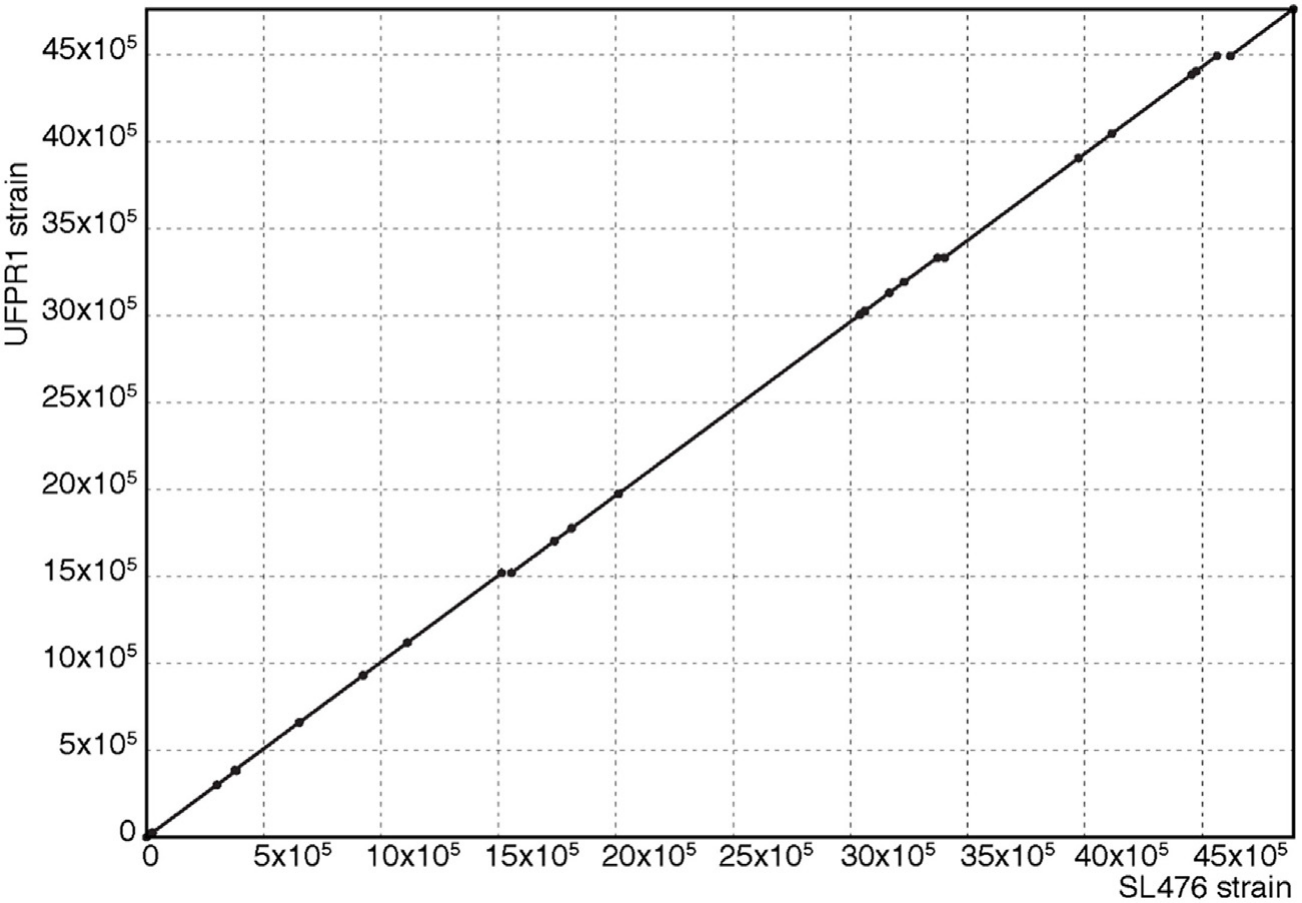
Alignment between genomic sequences from SL476 and UFPR1 strains. This dot-plot was generated with Mummer Software. The exact matches between genomic sequences are represented on the diagonal, showing the high conservation between the genomes with few missing fragments, as shown in Figure 6.

The whole genome of UFPR1 was sequenced to better understand the genotypic particularities of this strain and compared to the genomic sequence of a multidrug-resistant SH SL476 strain. As shown in Figure 6, the assembled genomic sequence from UFPR1 strain was 128 kb smaller than SH SL476 sequence, with important deletions of 11 chromosomal fragments in the Brazilian strain. Three of them were greater than 30, 40, and 50 kb, encompassing several important genes (Datasheet S1 in Supplementary Material). Genomic regions without similar sequences in the compared genome can be observed by red dashes in Figures 6A,B. Nevertheless, the comparison between the genomes of these strains revealed high similarity with few translocation events and conserved synteny (Figure 7). Moreover, no plasmid-sequences were found in the assembled sequences from reads of the UFPR1 strain (BioProject NCBI number PRJNA378710), using Canu software v1.3 (33) to correct all input data.

The genomic analysis of deleted regions revealed that 171 genes were present in SH SL476 strain but absent in UFPR1 strain (Datasheet S1 in Supplementary Material). The analyses of the 171 genes of SL476 showed that: (i) 46% were related to the codification of hypothetical proteins; (ii) 28% were mRNA sequences use to produce proteins with known functions involved in cell cycle regulation, DNA replication, virulence, drug resistance, and salt efflux; (iii) 16% correspond to encoded proteins related to the DNA recombination process (transposases and invertases genes), and (iv) 10% are encoded viral proteins (conjugal transfer, integrase, capsid, and tail proteins). Some of the genes in the absent regions were duplicated in SL476 genome or were located in another genomic region in UFPR1. Some genes were completely absent in UFPR1 strain (Datasheet S1 in Supplementary Material) such as (i) *aph3* and *aph*6 genes that codify two isoforms of aminoglycoside O-phosphotransferase proteins; (ii) *tem-1* gene that codifies a protein associated with an antibiotic resistance mechanism in bacteria; (iii) *qacE*Δ*1* gene involved in resistance to a large spectrum of quaternary ammonium compounds (QAC); (iv) *sul1* gene involved in the sulfonamide resistance; (v) *tetB* gene linked to the efflux of tetracycline, and (vi) *lysR* gene that codifies the transcriptional activator of *lysA* gene, which encodes the diaminopimelate decarboxylase involved in a lysine synthesis pathway. *LysR* gene belongs to the LYSR-type family transcriptional regulator, which regulates a varied set of genes involved in virulence, metabolism, quorum sensing, and motility (34). In the alignment of the genomes, the presence of five insertions was observed in the UFPR1 strain coding some genes like *bgt, bgr*, and *rpoS*. These genes are also present in the SL476 strain in other genomic regions and are correlated with important phenotypes found in UFPR1, such as virulence and organic acids resistance. Only five chromosomal fragments were found in UFPR1 compared to SL476 (Datasheet S2 in Supplementary Material); however, these fragments have been identified in several other strains demonstrating that they are not exclusive to UFPR1.

## Discussion

The results from the *in vivo* studies showed that SH UFPR1 strain do not seem to affect the zootechnical performance of broilers although these results should be taken with care given the fact that our experimental layout in terms of number of replicates was not designed to be highly sensible to changes in performance, but rather to be appropriate for all the other measurements described in this report.

No clinical signs were observed after SH UFPR1 oral challenge. Other studies have shown that non-typhoid *Salmonella* infection in chickens does not result in morbidity or severe clinical signs in spite of intestinal colonization, and liver and spleen bacterial infiltration (2, 3, 35). The SH UFPR1 strain infection produced mild histologic alterations in liver and cecum compared to non-challenged birds, mainly associated with inflammatory processes. No differences on IL-12 mRNA expression in liver on SH-challenged vs. non-challenged birds were observed as opposed to IL-10 mRNA liver expression which increased in the former group.

Shanmugasundaram et al. (36) showed that CD4+CD25+ (Treg) cells increase in number in the cecum of chickens infected with *Salmonella* Enteritidis. These cells collected from cecal tonsils of *S*. Enteritidis*-*infected birds and re-stimulated *in vitro* with *Salmonella* antigen had higher IL-10 mRNA content compared to those in the control group (*P* < 0.05). The CD4+CD25+ cells were associated with suppressing the immune response and maintaining the *Salmonella* infection in the host (4). The Treg cell was not marked in this study, but the absence of clinical signs, the apparent lack of effect on zootechnical parameters, along with the increase of IL-10 mRNA found in the present work suggest that this mechanism could be similar in the UFPR1 strain.

Associating phenotypic observations with genomic sequence information of an organism can contribute to understand some of its key biologic aspects, such as mechanisms for genetic information storage, genome organization, the effect of deletion, insertion, inversion, and translocation on the genome function. To that aim, the maximum number of possible genome sequences is necessary.

Whole-chromosome alignments made in the present genomic study showed that, besides phenotypic differences, the UFPR1 strain has a genome very similar to that of the multidrug resistance SH SL476 strain overall (Figure 6). However, several chromosomal fragments that harbor various important genes were lost in UFPR1 (Datasheet S1 in Supplementary Material). The *aph3* and *aph6* genes that were deleted in UFPR1, encode two isoforms of aminoglycoside O-phosphotransferase that participate in the primary mechanism of resistance to aminoglycosides, such as kanamycin, gentamycin, streptomycin, and neomycin. Both genes are frequently found together with transposable elements (37). The *tem-1* gene, which codifies the β-lactamase protein in bacteria, is associated with β-lactam antibiotic resistance and was also deleted in UFPR1. The protein produced by the translation of this mRNA fragment is able to hydrolyze penicillin and first-generation cephalosporin (38). Fragments of chromosome in which genes related to the production of proteins involved in DNA replication, such as DNA polymerase, DNA helicase, DNA resolvase, and DNA topoisomerase, were also found to be deleted in UFPR1 with copies of these genes present in other genomic DNA regions, indicating that their deletion did not affect UFPR1’s replication. In agreement with this observation, UFPR1 presented normal growth when cultured *in vivo* in the present report.

Likewise, deletion of *qacE*Δ*1, sul1*, and *tetB* genes were found in SH UFPR1 strain. The former gene has been associated to resistance against a large spectrum of cationic compounds such as intercalating dyes, diamidines, and biguanides (39), *sul1* to tolerance to sulfonamide (40) and *tetB* to tetracycline efflux (41). Although other genes linked to tetracycline tolerance were found in UFPR1 strain, such as *tetA* class B and *tetA* class C genes, the deletion of *tetB* gene could explain the intermediate tetracycline resistance observed in this report. The deletion of *LysR* gene in UFPR1 may be linked to its low pathogenicity since microorganisms lacking it have been found to be less virulent (34, 42).

Gene deletion is used as an evolutionary process in bacteria in which small genomes have evolved from large genomes, with natural selection acting as a significant driver of gene loss and reductive genome evolution (43). However, bacteria genome could be increased by the acquisition of genetic fragments transferred horizontally (44).

Interestingly, no sequence from plasmids among those assembled was observed. Genome stability is threatened by transposons, which are able to create repetitive sequence islands that can initiate ectopic recombination (45). The present results showed that UFPR1 has several deletions of genes of various transposases suggesting that DNA transposition could be decreased in this strain. The UFPR1 genome presented five different fragments that are absent in SL476 but present in other *Salmonella* genomes evidencing the fact that they are not exclusively of UFPR1. In those five fragments, important genes related to some of the phenotypic characteristics reported herein in UFPR1 were found, such as the *Bgt* and *Bgr* genes that are related to serotype transformation; and the *rpoS* gene, linked to sensitivity to lower temperatures. The proteins produced by the expression of *Bgt* and *Bgr* genes are related to the glucosylation of the O-antigen repeated units of lipopolysaccharide (LPS) and are correlated to serotype conversion in *Shigella flexneri* and also in *Salmonella* (46). The presence of this gene is also correlated to the increase of virulence and resistance to oxidative stress (47).

Activation of *rpoS* gene is involved in cold sensitivity of *Salmonella enterica* serovar Typhimurium (48). The *rpoS* gene codifies for an alternative sigma factor that regulates many cellular responses to environmental conditions, such as heat, alkaline, and acid stress. Mutations of this gene have been detected in pathogenic bacteria (49). Bacteria are subjected to acid stress situations such as to the extreme low pH of the stomach and to the organic acids that are produced in large quantities in the gut including acetic, propionic, and butyric acids. In both situations, bacteria activates mechanisms for acid tolerance response (ATR), for which *rpoS* is a key regulator, thus minimizing the lethal effects of the acid stress. However, *Salmonella rpoS* mutant fails to provide the same level of protection when compared to a wild-type strain. Therefore, *rpoS* mutant is ineffective to sustain the ATR resulting in rapid cell death when exposed to pH 3.0 (50, 51). The product of the *rpoS* gene regulates the virulence gene expression in *Salmonella* Typhimurium in response to conditions encountered in the host tissue. Mutations in the *rpoS* gene yield that bacteria unable to develop a complete ATR significantly reducing its virulence potential (51). The presence of *rpoS* gene in the UFPR1 strain could be involved to the resistance to SCOA found. It has been observed that the alternative sigma factor clearly plays an important role in protecting *Salmonella enterica* serovar Typhimurium against weak acids (52).

In a recent study, Dhanani et al. (15) demonstrated in an *in vivo* experiment that the resistant genes found in SL476 may explain its pathogenicity, colonization ability and persistence in chickens. The absence of several genes involved in tolerance to antibiotics and in the efflux of salt reported in this paper, could explain why UFPR1 was in general susceptible to antibiotics, but resistant to SCOA likely due to the presence of *rpoS* gene.

The use of SCOA can be effective for the control of *Salmonella* in broilers being an important tool for the poultry industry (6, 21). However, testing for the presence of the ATR gene in *Salmonella* strains could help avoiding the misuse of these substances.

In this report, we used comparative genomics to study some of the genotypic peculiarities of UFPR1 linking those findings to phenotypic observations such as its tolerance to SCOA and sensitivity to various antibiotics. The comparison of several genome sequences could reveal important aspects of the evolution of the different *Salmonella* strains, and in a more accurate analysis, help identifying single nucleotide polymorphisms involved in potentially unknown pathways that could be relevant to the study of the metabolism of *Salmonella* and its control. Our findings can also help developing effective strategies to control this agent in broilers, thus preventing food-borne disease in humans.

Salmonellosis causes great economic damage to the poultry industry worldwide from strains that are either pathogenic and non-pathogenic to humans. Developing methodologies that reliably and promptly differentiate pathogenic from non-pathogenic strains could ameliorate that economic loss.

## Conclusion

The infection of *Salmonella enterica* serovar Heildelberg UFPR1 in broilers did not affect zootechnical performance and promoted a mild inflammatory reaction on cecum and liver.

The use of different SCOA in drinking water or feed was ineffective against *Salmonella enterica* serovar Heildelberg UFPR1 strain in the present layout.

The genomic findings showed several differences between SH UFPR1 strain and the pathogenic SL476 strain.

The absence of several genes involved in antibiotics resistance and in salt efflux; along with the presence of *rpoS* gene, could explain the overall high susceptibility of UFPR1 to the antibiotics tested, and its resistance to SCOA. This information can help the poultry industry on designing SH control programs targeted against this specific strain.

Understanding the phenotypic and genotypic differences among *Salmonella* strains could help improving our knowledge on their metabolism, which could ultimately lead to their effective control.

## Ethics Statement

The experiments were approved by the Ethical Committee of Agricultural Sector of Federal University of Parana under approval number: 037/2016 and 014/2016.

## Author Contributions

ES—isolation of *Salmonella* Heildelber strain UFPR1, design *in vivo* and *in vitro* trial, analysis and interpretation of data, drafting and revising it critically, and final approval of the version. RH—design *in vivo* and *in vitro* trial, analysis of data, drafting the work and revising it critically, and final approval of the version. JW—carry on the *in vivo* and *in vitro* trial, analysis of data, drafting the work and revising it critically, and final approval of the version. RG-E—design *in vivo* and *in vitro* trial, analysis, interpretation of data for the work, drafting the work, and final approval of the version. MC, CF, PM, AC—analysis, interpretation of genomic data and revising it critically, and final approval of the final version.

## Conflict of Interest Statement

The authors declare that the research was conducted in the absence of any commercial or financial relationships that could be construed as a potential conflict of interest. The handling editor and reviewer KG declared their shared affiliation and involvement as co-editors in the Research Topic.
